# Second MRSA outbreak in a nursing home; role for a super spreader or super sensitive resident

**DOI:** 10.1186/1753-6561-5-S6-P162

**Published:** 2011-06-29

**Authors:** P Gruteke, A Haenen, M Scholing, C van Teunenbroek, A de Neeling

**Affiliations:** 1 OLVG, Amsterdam, Netherlands; 2 RIVM, Bilthoven, Netherlands; 3 Zorggroep, Almere, Netherlands

## Introduction / objectives

MRSA prevalence is low in nursing homes (NH) in the Netherlands. However, outbreaks in NH are usually larger than those occurring in hospitals. The national MRSA database shows that in the last three years 24% of the outbreaks occurred in NH. The NH under investigation experienced outbreaks in 2007 and 2010. Two residents and one health care worker (HCW) were involved in both outbreaks. One of these residents (R-Z) has diabetes and pressure ulcers, and wasÂ therefore not considered for eradication therapy. R-Z could have acted as a super spreader.

## Methods

Laboratory records and outbreak courses were studied for the role of R-Z in both outbreaks.

## Results

The first outbreak (MRSA spa t032) was noted when two residents were admitted to the nearby hospital, and gave rise to spread to two hospital patients and two nurses. 12 NH residents and 4 NH-HCWs were found colonized. The second outbreak (MRSA spa t539) was noted in the OPD of the hospital without further colonization’s in the hospital. 11 NH residents and 5 NH-HCWs were colonized in the second episode. During 2009 and early 2010 R-Z had been tested negative for MRSA on 7 occasions. Precaution measures instituted in 2007, were lifted. A positive MRSA result in May 2010 was initially mistaken as a recurrence, and neighboring residents and attending HCWs wereÂ screened negative. Only after the outbreak was noted 2 months later, typing revealed colonization with a new strain.

## Conclusion

We cannot conclude that persistent super spreading occurred, since R-Z eventually cleared the MRSA. The fact that R-Z was the first to get colonized with the new strain illustrates increased susceptibility. Colonization-prone patients may require life long protective measures and surveillance.

## Disclosure of interest

None declared.

